# Supplementary material for: Iodine Bioavailability and Accumulation of Arsenic and Cadmium in Rats Fed Sugar Kelp (Saccharina latissima)
Source: Foods. 2022 Dec 7;11(24):3943. doi: 10.3390/foods11243943 (PMC9777903; doi:10.3390/foods11243943)
Supplement: Supplementary file 1 [file foods-11-03943-s001.zip › foods-1997962-supplementary.pdf]

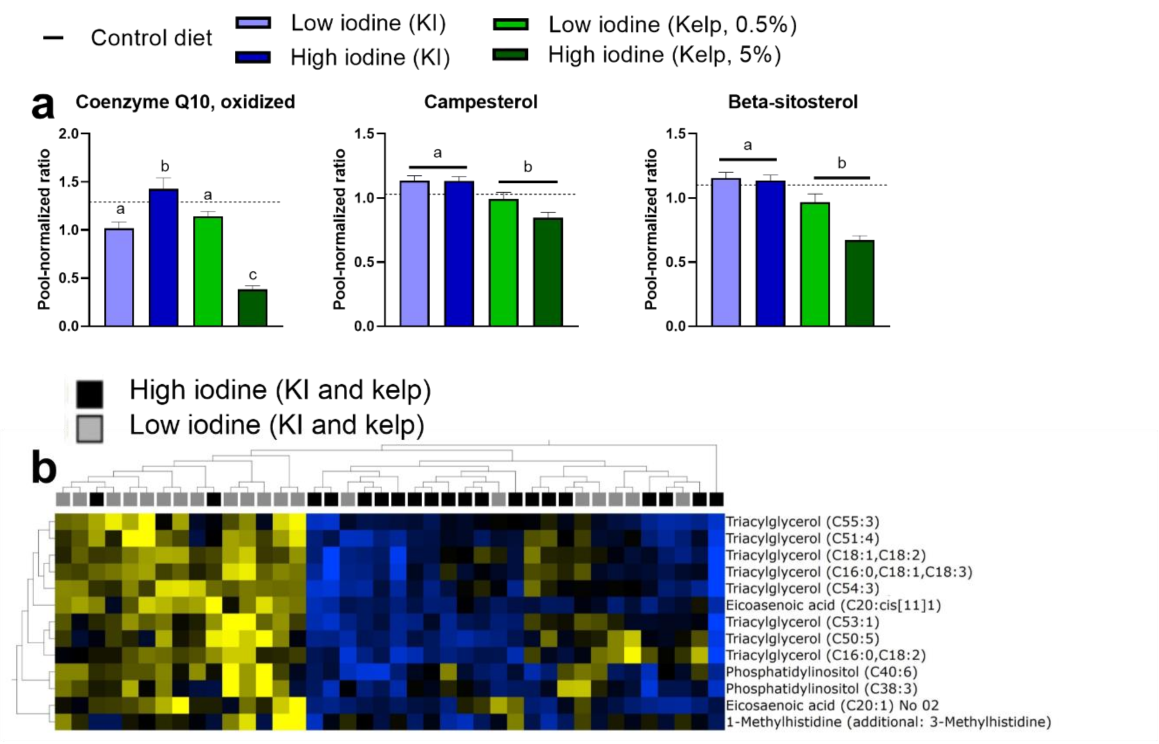

Figure S1. (a) Liver metabolites regulated in rats fed low and high KI and sugar kelp diets. (b) Liver metabolites regulated comparing low and high iodine diets, independent of iodine sources. Measured levels were semi-quantitative and normalized against a pool reference sample (n = 9–10). Different letters denote significant differences ($P < 0.05$) between the iodine sources, where # indicates a significant difference between the iodine doses (independent of source).
